# Unravelling the quality of malaria microscopy across Kinshasa, DR Congo

**DOI:** 10.5281/zenodo.10630995

**Published:** 2024-02-09

**Authors:** Pierre Mukadi-Kaningu, Fortunat Kandanda Muele, Nestor Tshimanga, Joel Unandu, Brigitte Mbuyam-ba Mbamba, Eric Mukomena Sompwe

**Affiliations:** 1 Université Pédagogique Nationale, B.P. 8815, Kinshasa 1, DR Congo.; 2 Institut National de Recherche Biomédicale, Kinshasa, DR Congo.; 3 Santé Rurale, 76, avenue de la Justice, Gombe-Kinshasa, DR Congo.; 4 Programme National de Lutte contre le Paludisme, Kinshasa Kintambo, DR Congo.

## Abstract

**Introduction:**

In the current study we assessed clinical laboratories’ staff ability across the city of Kinshasa with particular focus on their practices and performance regarding malaria microscopy.

**Materials and Methods:**

This was a non-random cross-sectional study included clinical laboratories in Kinshasa and focused on cross-checking of blood slides, a questionnaire and checklist according to standardised analytic malaria microscopy procedures. Regarding the cross-checking of slides, participant responses were considered ‘corrects’ in cases of complete congruence with the reference; ‘acceptable’ for malaria-positive slides but no identification of *Plasmodium* species, stage of development, parasite density and/or reported as *P. falciparum* instead of ‘P. non falciparum’; and ‘incorrect’ if ‘false positive’ and ‘false negative’ cases.

**Results:**

Eighty-eight among the 90 targeted clinical laboratories (participation 97.8%) took part in the investigation from February to July 2019. The ability assessment revealed that individuals qualified to perform thick blood films (TBF) according to the national malaria control program (NMCP) procedures ranged from 48.6% to 100.0%. Overall cross-checking performance of 167 eligible routine slides was relatively low: 37.7%; 25.8% and 36.5% of correct, acceptable and incorrect responses, respectively. The first routine slide was correctly and acceptably scored respectively by 35.3% and 28.2% of participating laboratories (n = 85); and the second, by 40.2% and 23.2% respectively (n = 82). The sensitivity and specificity were found to be 79.4% and 53.8%, respectively. However, the relative high scores reported in relation with the ability needed to perform TBF based on NMCP standards contrasted with the poor performance from cross-checking slides. Consecutively, only one-third of the 88 participating laboratories reached a score > 60% in agreement with NMCP procedures and had acceptable responses to cross-checked slides.

**Conclusions:**

The study was conducted as part of the activities relating to "Ensuring early diagnosis and prompt malaria treatment" component of the national malaria control strategy with NMCP support. More laboratories must implement clear and standardised malaria microscopy procedures, and need to include more rigorous quality control.

## Introduction

Malaria remains a major public health challenge, particularly in resource-limited settings in sub-Saharan African, such as in the Democratic Republic of Congo (DRC), which bears one of the highest malaria burdens in the world (12.3%) and where 30 million cases and 80,000 deaths were reported in 2021 [1]. Challenging socio-economic conditions hinder the provision of critical care based on artemisinin-based combination therapy (ACT), adopted around the world, for malaria treatment that should be based on parasitological diagnosis [2]. Malaria rapid diagnostic tests, which are increasingly used in the DRC, are vulnerable to errors, especially due to HRP-2 antigen deletions and the failure to identify non-*Plasmodium falciparum* infections [3].

Malaria microscopy therefore remains the gold standard and is highly recommended by the World Health Organization (WHO) as well as the national malaria control programme (NMCP) of the DRC. Current national strategies for the supervision of staff performing microscopy are based on the organisation of the Congolese health system as recommended by WHO, i.e., cross-checking of slides and on-site evaluation are carried out by the national reference laboratory at the intermediate level laboratories, and by the latter on the peripheral level laboratories [4].

Furthermore, the Thick Blood Film (TBF) is one the most viable amongst analytical methods whose standard operating procedure (SOP) is validated by WHO. It is worth mentioning that this SOP allows laboratory staff to perform and then adequately stain a TBF and thin blood film (tBF) to detect blood parasites, including *Plasmodium* spp. [4]. In addition, TBF and tBF are commonly used to identify *Plasmodium* species and also estimate parasite density in the case of *P. falciparum* (Pf). To date the available literature on *Plasmodium* species allows relatively objective choice of appropriate antimalarial drugs, whilst parasite density remains necessary to assess malaria severity and parasite load during treatment [2].

However, TBF is often poorly performed in endemic and resource-limited settings such as in Kinshasa, the principal township of DRC, where it is commonly performed at various health facilities [57]. Of note, malaria prevalence in Kinshasa, around 23.0%, is similar to that observed nationwide. Poor performance of TBF is due to insufficient maintenance of microscope equipment, poor-quality stains, lack of training or quality assurance [8-9]. As a consequence, malaria misdiagnosis can affect case management which increases the risk of drug resistance development as well as the malaria burden overall in terms of morbidity and mortality.

The latest related studies among DRC clinical laboratories demonstrated poor performance, mainly in identifying *Plasmodium* species and estimating parasite density. Apart from assessing the quality of routine TBF amongst participating laboratories, these studies had not assessed pre-analytical and post-analytical steps in real time [7,9].

Therefore, this study assessed the ability of laboratory technicians to perform TBF in clinical laboratories in Kinshasa and their performance in examining their own routine TBF and tBF through cross-checking. The practice required strict monitoring by national-level microscopic experts according to WHO recommendation. To our best knowledge, this is the first study in DRC that simultaneously assessed the ability to perform microscopy and the performance in reading and interpreting TBF through cross-checking. The purpose was to contribute to the improvement of malaria microscopy practice for better case management.

## Materials and Methods

### Design

This non-random cross-sectional study was carried out jointly by the Université Pédagogique Nationale (UPN) and the NMCP; technical aspects were provided by Institut National de Recherche Biomédicale (INRB) which is a national malaria reference laboratory. The study was conducted from February to July 2019 in 88 clinical laboratories across Kinshasa, the capital city of the DRC widely populated with approximately 12 million citizens [5].

The study was conducted as part of the activities relating to ‘Ensuring early diagnosis and prompt malaria treatment’ component of the global malaria control strategy with support from the NMCP. The results had also been used for writing-up of dissertations in the Department of Medical Biology of the UPN.

### Participants

Sampling was exhaustive and non-random. Participating clinical laboratories, from 14 health zones of Kinshasa (n = 35), were members of the network of INRB which was supervised by the PNLP experts. They were selected based on their physical accessibility, convenience, availability, free consent and were categorised as Referral hospital, Health Centre, Clinic, or public/private specialised laboratory.

### Questionnaire and checklist

A questionnaire was conceived based on previous studies and consisted of closed and open questions. It was filled in by participating laboratories before the assessment. Questions addressed health facility type, any malaria trainings, laboratory workload (including microscopy and malaria rapid diagnostic test) and tests positivity rate, microscope maintenance and reagent management, internal quality control (IQC) and external quality assessment (EQA) [4,9].

The checklist was designed in accordance with the TBF SOP used and recommended by the national malaria reference laboratory. Assessment addressed blood sampling, preparation of TBF and tBF, staining, microscopic reading, interpretation, and result reporting [10]. It was completed by assessor during on-site assessment. Both documents (questionnaire and checklist) were validated through previous studies and surveys [4,9].

### Study procedure

The survey and collection of routine TBFs to be rechecked in the reference laboratory was carried out by three assessors who were medical biology trainees at the Université Pédagogique Nationale. They were trained on the performing of TBF in accordance with the SOP from PNLP and on the methodology for steering the study. Ability assessment to perform TBF among participating clinical laboratories was conducted during opening hours (8:30 AM - 4:00 PM). Assessors visited laboratories and had a meeting with the laboratory responsible to explain the objectives and procedures to be implemented during the study with aim to obtain study consent. Consecutively, the laboratory responsible scored the questionnaire and based on a checklist the assessor evaluated the ability of technical staff in charge of TBF, starting from pre-analytical to post-analytical phase. Assessment consisted of observation and short questions, if necessary, to the technical staff during the process of TBF performance. As a last step in the process, two TBF were randomly selected from routine slides performed during the day of the survey: these consisted of one positive slide (for *Plasmodium* spp.) and one negative slide as reported by the participating laboratory through its register book. Both these TBF, labelled as TBF-1 and TBF-2 for each participating laboratory, were sent to the INRB laboratory for blinded re-checking by national-level microscopy experts. Finally, the results of 2 routine TBFs, completed questionnaire and checklist were packaged and sent to the principal investigator.

### Data entry and analysis

Results from cross-checking slides, answers to the questionnaires and checklists were compiled into an Excel spreadsheet (Microsoft Corporation, Redmond, Washington, USA). In order to effectively assess technical staff’s ability to perform TBF, particular scores were assigned to each step of the procedure corresponding to a question of the checklist. The scores ‘1’ and ‘0,5’ were given for procedural step that has a direct impact on the final TBF result and the relative step, respectively. For example, ‘Identifying TBF slide’ was scored ‘1’ while ‘Put on gloves’ was scored ‘0,5’. Indeed, if the TBF slide is not identified or misidentified, it will be impossible to know to which patient the result is attributed. However, to wear gloves or not does not have a direct impact on the final result, but the non-use of gloves constitutes a biosafety gap for the staff.

Routine TBF responses were compared to those of the national malaria reference laboratory as ‘correct’, ‘acceptable’, or ‘incorrect’. Participant's responses were considered to be correct in case of complete compliance with the reference. Acceptable responses were cases related to positive TBF for *Plasmodium* spp., which did not report the *Plas-modium* species, stage of development, parasite density and/or reported ‘*P. falciparum*’ instead of P. non-falciparum. Incorrect results were those that negatively impact clinical status of the patient, i.e., ‘false positive’ and ‘false negative’ results. It should be noted that the distinction between ‘acceptable’ and ‘incorrect’ found its essence on the potential effect the error could have on the patient’s diagnosis and clinical management. The best ability and performance of each participating laboratory was the highest score needed to perform TBF and get relatively correct results from cross-checking routine TBFs.

Instead, comparison of scores amongst participating laboratories was based on the relevant factors pre-defined in the questionnaire, and was further compared to those reported in previously published reports [9]. Differences in proportions were tested for significance using the Fischer's exact, Kruskal-Wallis and Mann-Whitney U tests, with a p-value ≤ 0.05 was considered statistically signifcant.

### Ethics Statement

Participation in the study was free and voluntary; informed consent was obtained prior to inclusion in the study. The identity of the study participants was encoded and the code was only known by the study coordinator. The study protocol was approved by the institutional review board of the University of Lubumbashi (UNILU/CEM/ 097/2019).

## Results

A total of 88 out of 90 targeted clinical laboratories participated in the study, giving a participation rate of 97.8%. Five levels of participating laboratories (n = 88) were considered: Referral hospital’ (15.9%), private facilities (22.7%), clinics (20.5%), specialised laboratories (2.3%) and health centres (38.6%). The relative coverage of the city of Kinshasa was found to be 50.0% and 29.8% for referral hospitals and health centres, respectively, considering that only 14 out of 24 municipalities were investigated (Table 1). The study revealed that participating laboratories were mostly managed by laboratory technicians and medical biologists (94.3%, n = 88).

**Table 1.** Characteristics of the 88 health facilities that participated in the study, and questionnaire answers.

	Specialised laboratories (n=2)	Referral hospitals (n=14)	Clinics (n=18)	Health centres (n=34)	Private facilities (n=20)	Total (n=88)
	Specialised laboratories (n=2)	Referral hospitals (n=14)	Clinics (n=18)	Health centres (n=34)	Private facilities (n=20)	Total (n=88)
SOP microscopy available (%)	100,0	100,0	83,3	91,2	80,0	88,6
Internal Quality Control implemented (%)	100,0	85,7	61,1	41,2	55,0	56,8
Participation in EQA (%)	50,0	50,0	50,0	29,4	30,0	37,5
Malaria RDT in use (%)	50,0	92,2	83,3	100,0	85,0	90,9
*mRDT brand(s) in use (%)*						
Care start	0,0	28,6	27,8	55,9	30,0	38,6
SD Bioline	50,0	50,0	50,0	32,4	45,0	42,0
Care start + SD Bioline	0,0	14,3	5,6	11,8	10,0	10,2
*Participation in training (%)*						
Yes	100,0	92,9	72,2	97,1	90,0	89,8
<2 years^3^	50,0	64,3	38,9	38,2	50,0	45,5
*Other data*						
TBF /year, median (IQR)^b^	7431	4412	5400	6800	4673	5998
	(4246-10615)	(2880-7654)	(3116-14700)	(1825-11500)	(1800-13550)	(1875-12038)
Positivity rate of TBF (%), average (SD)^c^	63,7	53,6	47,3	52,2	52,5	51,4
	(43,1-84,3)	(33,1-74,1)	(24,2-70,4)	(35,2-69,2)	(34,9-70,1)	(32,2-70,6)
mRDT/year, median (ICR)^d^	11600^f^	2965	4500	7670	7600	4155
		(1535-5485)	(1415-11850)	(1084-10350)	(476-14160)	(1083-11675)
Positivity rate of mRDT (%), average (SD)	65,5^f^	40,8	45,7	52,2	47,2	48,3
		(17,9-63,7)	(26,6-66,8)	(38,0-66,4)	(32,8-61,6)	(31,2-65,4)

SOP= standard operational procedure; EQA= external quality assessment; mRDT= malaria rapid diagnostic test; TBF= thick blood film; IQR= interquartile; SD= standard deviation. a: Training in the past two years; b: Number of TBF performed in 2018; c In 2018; d: Number of mRDT performed in 2018; e: In 2018; f: Data collected for one participant only.

### Ability to carry out the thick blood film

Out of 88 individual technicians assessed, reported scores ranged from 48,6 to 100,0% (Table 2). Moreover, it was found that of those who had implemented a standard operating procedure for malaria microscopy, 14,8% and 81,8% had reached a score of 100% and between 65,0 and 100,0% respectively (n = 88).

**Table 2.** Ability of individuals in participating laboratories to perform thick blood film; results from on-site assessments (n = 88).

	Specialised laboratories (n=2)	Referral hospitals (n=14)	Clinics (n=18)	Health centres (n=34)	Private facilities (n=20)	Total (n=88)
Collected the necessary materials for blood collection	100,0	92,9	88,9	82,4	90,0	87,5
Wore gloves	100,0	71,4	72,2	67,6	60,0	68,2
Slide identified	100,0	100,0	100,0	97,1	100,0	98,9
Disinfected the sampling site	100,0	100,0	94,4	100,0	100,0	98,9
Let the disinfectant dry	100,0	92,9	77,8	88,2	85,0	86,4
Used single-use needle	100,0	100,0	100,0	100,0	100,0	100,0
Discarded the lancet in sharps box	100,0	92,9	83,3	76,5	80,0	81,8
Collected the necessary materials for staining and microscopy	100,0	100,0	77,8	67,6	85,0	79,5
Good preparation of smear*	100,0	85,7	94,4	94,1	90,0	92,0
TBF and tBF] on the same slide	100,0	42,9	44,4	32,4	15,0	34,1
Work solution prepared correctly	100,0	42,9	44,4	32,4	15,0	34,1
Correct staining	100,0	100,0	100,0	91,2	95,0	95,5
Time of staining respected	100,0	92,9	100,0	97,1	100,0	97,7
Slide dried before reading	100,0	100,0	100,0	94,1	95,0	96,6
Slide read for 10 min before declaring negative	50,0	100,0	88,9	94,1	80,0	89,8
Using a good microscope	100,0	100,0	100,0	82,4	80,0	88,6
If TBF positive, identified Plasmodium spp.	50,0	28,6	50,0	35,3	20,0	34,1
If TBF positive, counted asexual parasites	0,0	42,9	33,3	17,6	15,0	23,9
Correctly registered results	50,0	35,7	38,9	14,7	15,0	23,9
Used a specific register	100,0	100,0	100,0	100,0	95,0	98,9
Overall scores						
<50%	0,0	0,0	0,0	2,9	0,0	
50-70%	0,0	7,1	16,7	23,5	35,0	
70-99%	100,0	64,3	61,1	61,8	60,0	
100%	0,0	28,6	22,2	11,8	5,0	

* Criteria previously validated were considered: State of slide (new or reused), dimension and thickness, intactness of TBF, haemolysis of the red blood cells, staining and distribution of white blood cells, if TBF was positive (differential staining between ***Plasmodium*** nucleus and cytoplasm) and the presence of Giemsa stain precipitates).

Key steps of the TBF analytical procedure were performed efficiently by (i) 87,5% having collected the necessary material needed for blood collection; (ii) 81,8% having discarded the lancet in the sharps box immediately after finger pricking; (iii) 34,1% having carried out tBF and TBF; (iv) 96,6% having adequately prepared a Giemsa stain working solution; (v) 95,5% and 97,7% having covered the whole preparation with the Giemsa stain and effectively observed the staining time, respectively; (vi) 96,6% having allowed the slide to dry in open air for the prescribed duration before microscopic reading (minimum 3 minutes) and 88,6% having used a good microscope and having a 100x objective in good condition; (vii) 89,8% having microscopy examined TBF for at least 10 minutes before declaring it ‘negative’; (viii) 34,1% having identified the *Plasmodium* species in case of TBF positive; (ix) 23,9% having counted the asexual parasites in case of *P. falciparum* and (x) 23,9% having correctly noted the results (such as ‘negative’ or ‘positive’, if TBF positive: ‘*Plasmodium* species and number of asexual parasites per μl of blood’). Out of 30 participants who identified the *Plasmodium* species, 20,0% did not use the tBF as SOP-recommended, therefore leading to misidentification (Table 2).

### Results of cross-checking slides

**Figure 1.**
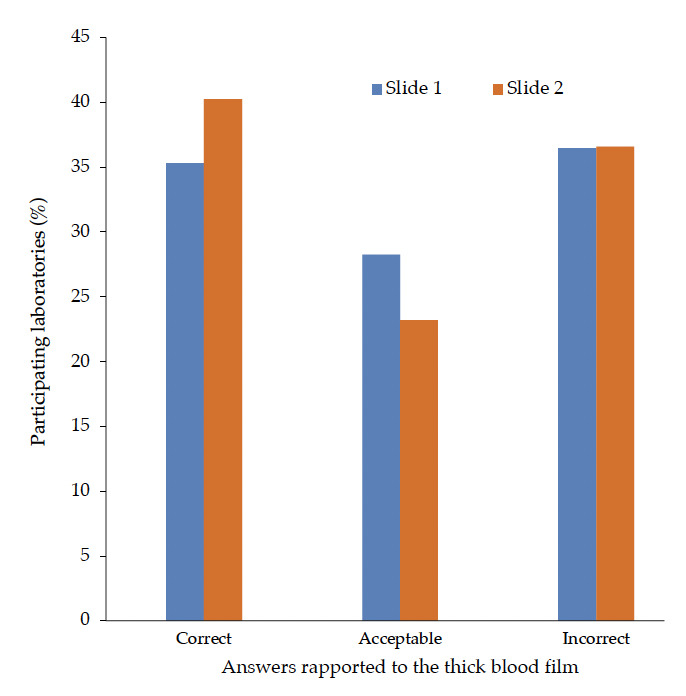
Proportion (%) of correct, acceptable and incorrect responses to the two reference slides (n = 167) . Correct: answers fully comply with the reference; Acceptable: TBF positive for *Plasmodium* spp. but no species identification, no development stage, no parasite density and/or *P. falciparum* instead of P. non falciparum; Incorrect: could have negative impact on the patient and clinical management; for instance, ‘false positive’ or ‘false negative’ results.

A total of 172 out of the anticipated 176 slides were collected: one hundred were positives for *Plasmodi-um* spp. and 72 contained no parasites, according to the participants' reports. However, based on rigorous checking by experts from the referral laboratory, five slides were rejected because they did not contain smears. Finally, 167 slides were eligible and were considered for further analysis among which 63 were positive for *Plasmodium* spp. and 104 did not contain any parasites. Figure 1 shows proportions of laboratories reporting ‘correct’, ‘acceptable’ and ‘incorrect’ responses as compared to the reference responses based on TBF1 and TBF2.

In addition, 63 (37,7%), 43 (25,7%) and 61 (36,5%) reported responses were found to be correct, acceptable and incorrect respectively (n = 167). Moreover, out of 50 correct responses to positive TBF, *Plasmodium* spp. identification and parasite density represented 18,0% and 55,6%, respectively. False negative and false positive results represented 7,8% and 28,7% respectively (n = 167).

Overall, 35,2% (n = 88) of the participating laboratories scored >60% to perform TBF and achieved correct and acceptable responses to cross-checking slides. Among 17,1% (n = 88) of participants with incorrect responses for cross-checking slides, only 2 of scored < 60% for carrying out a TBF.

Regarding their analytical performance, sensitivity, specificity and positive and negative predictive values were 79,4%, 53,8%, 51,0% and 81,2% respectively, as compared to reference responses.

### Questionnaire results

The median age of individual participant-technicians was 33 years (interquartile range, IQR: 29-38) and the median time of malaria microscopy’ experience was 5 years (IQR: 3-7). The sex ratio was 1,4 for males.

Table 1 shows the characteristics of 88 health facilities and data concerning malaria RDT used and malaria microscopy good practices, including training, workload, internal quality control (IQC) and external quality assessment (EQA). Out of 88 laboratories that responded to the questionnaire, all participants performed TBF and 90,1% of them also performed malaria rapid diagnostic tests (RDT). A proportion of 89,8% had received training in malaria diagnosis; however, only half of them (50,6%) had been trained in two years. It was found that a total of 88,7% had the malaria microscopy SOP as recommended by the NMCP. Internal quality control was carried out by 56,8% (n=88) of participants as follows: (i) through agreement of the results of approximately 2 and 3 technicians (68,0%, n=50) and (ii) the use of control slides (28,0%, n=50). It is noted that 43,2% (n=88) had not implemented IQC. For instance, laboratories that participated in the malaria diagnosis EQA nationwide between 2010-2014 represented 37,5%.

**Figure 2.**
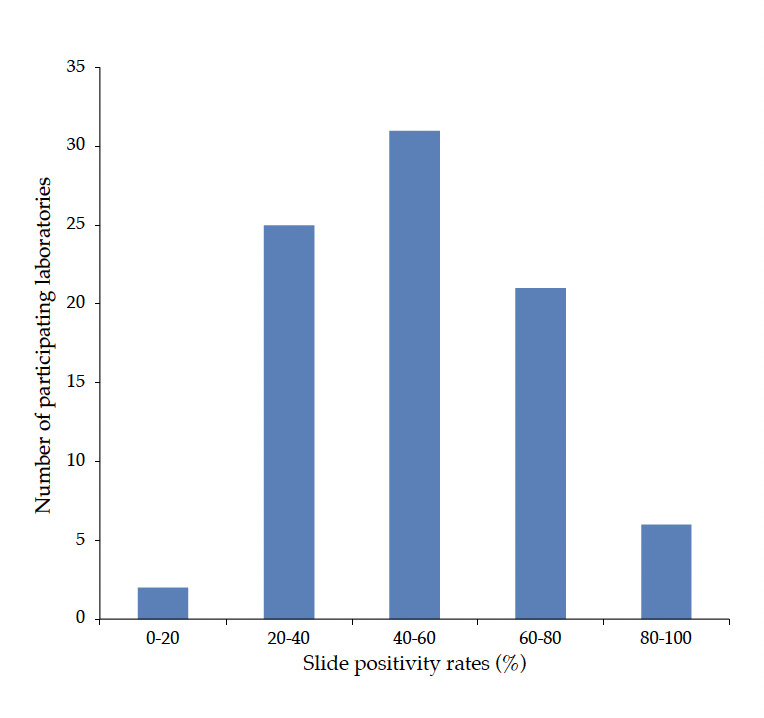
Reported slide positivity rates for the participating laboratories (n = 85).

The average of TBF positivity rate was 51,4% (32.2-70.6); but 2 (2,4%), 25 (29,4%), and 58 (68,3%) of the participants reported TBF positivity rates of <20%, 20-40%, and >40%, respectively; and 2 (2.4%) participants reported positivity rates of >90% (Fig. 2). During the previous investigation all participants used Giemsa stain and procured it from private suppliers (61,4%) and private vendors who visited and delivered the product on-site (26,1%). As a result, Giemsa stain as a stock solution was the most commonly supplied; to 81,8% of them.

It is worth noting that malaria RDT brands used were those recommended by the NMCP, specifically Care Start and SD Bioline.

## Discussion

Based of the above findings, the ability to perform TBF and the performance of participant laboratories in cross-checking slides were good and poor, respectively. Moreover, the TBF SOP was implemented by the majority of staff in the laboratories assessed. As a result, scores of 100% and > 50,0% were achieved by 14,8% and 92,0% of individual participants, respectively (n = 88). Nevertheless, 36,5% of the responses reported from cross-checking slides were incorrect. As a result of this, sensitivity and specificity were found to be 79,4% and 53,8%, respectively, as compared to referral responses.

It should be noted that the relatively encouraging scores achieved during the study contrasted with the poor performance in cross-checking slides. The investigation revealed that only one third of 88 participating laboratories who achieved a score >60% according to the SOP of TBF had returned correct and acceptable cross-checking responses.

Although relatively high scores were obtained in performing TBF (>50% for 92,0% of participants), several steps of the TBF SOP were inappropriately performed. Moreover, the findings revealed that more than one-tenth of participants had not previously collected the necessary material for blood collection, preparation and staining of blood films; this inattentiveness may find its origin in mishandling the process. These include (i) detachment of smear film during staining when the slide has not been degreased and (ii) thickening of the blood prior to defibrillation during smear preparation. In this particular case, smears will not be stained with Giemsa because blood cells are trapped into the fibrin. This error often leads to a false negative result due to non-haemolysis of red blood cells [4]. Although it has no impact on the final result of the TBF, unavailability of a sharps box in one-fifth of the laboratories constitutes a major biosafety non-compliance [10].

Double smearing (TBF and tBF on the same slide) is recommended by WHO and the NMCP to corroborate a positive result with species of *Plas-modium* and parasite density [2,11]. Only one third of the participants had performed double smearing, which explains the non-identification of the *Plasmodium* species by ¾ of participants. In addition, absence or misidentification of *Plasmodium* species, reported by >65.0% of participants in this study, is common in the DRC [6,9,12,13]. Although insignificant (6,8%, n = 88), the proportion of participants who identified the *Plasmodium* species without having made a tBF is of critical concern.

A third of participating laboratories did not regularly maintain their microscopes. But more than one-tenth did not have good microscopes; the 100x objective was lacking. Although some recent studies point to new technologies, microscopy is still the gold standard for malaria diagnosis [14]. Like quality reagents and the microscopist's expertise, the microscope must receive routine care and preventive maintenance as described in previously published guides [15].

On the other hand, despite the recommendation of the WHO and the NMCP, as well as several trainings provided and EQA sessions, reporting of TBF results were not compliant in ¾ of cases. Indeed, the *Plasmodium* species was reported by only one third of the participants. Also, despite the identification of *P. falciparum*, parasite density was not commonly determined [4,10]. It is worth mentioning that to the best of our knowledge few studies have evaluated the reporting of TBF results in DRC or elsewhere [6-9].

More than ¾ of participants did not estimate parasite density. Moreover, these proportions were much higher than the 31,8% reported in Ethiopia, but were overall similar to those reported in endemic settings and particularly in DRC [9,13,16]. It is usually difficult to assess the factors involved in the failure to achieve parasite density. Firstly, the clinician would neglect this parameter which is nevertheless essential in the classification of malaria (non-complicated or severe) and in the monitoring and evaluation of the efficacy of antimalarial treatment. Secondly, the workload and especially the lack of motivation or training of laboratory technicians could be the origin of the deficiency encountered during the investigation.

Inter-laboratory comparison programmes, such as cross-checking routine blood slides, are likely to reveal inadequacies in the accuracy of results and the performance of a specific laboratory due to poor infrastructure and equipment, poor quality of reagents, unskilful staff or poor day-to-day practices [4,17].

With 35,2% (n = 88) acceptable responses, although similar to the 35,0% (n = 277) and 29,3% (n = 400) previously reported in the DRC, the overall performance of participating laboratories in the cross-checking routine slides in this study was poor as compared to those reported elsewhere (for instance, 93.8% in Ethiopia and >99% in Malaysia and Senegal) [6,18,19].

The study shows that the main errors reported were (i) non-identification of *P. falciparum*, (ii) non-estimating parasite density, and (iii) false positive results. Indeed, proportions of responses related to *P. falciparum* identification (18,0%) and estimating parasite density were very low compared to all reports published from endemic areas [6-7,9,18-19]. Moreover, the proportion of false positive results observed in this study (28,7%) was within the range reported until recently from the African region, including DRC (between 1 and 51%) [6,8,17,20-22].

Despite a relatively high attendance in the training, the sensitivity (79,4%) and specificity (53,4%) reported in this study remained lower than 90 and 80% respectively, as per WHO recommendations [4]. However, considerably different and better performance was reported elsewhere: sensitivity and specificity of 97,8 and 98,2% respectively in Senegal, and 50,0 and 70,6% in Nigeria [6,23]. The findings highlight the need for rigorous training of not less than one week per year in order to significantly improve and maintain the abilities of microscopists for the detection of blood parasites, identification of *Plasmodium* species, particularly *P. falciparum*, and parasite density estimation [24].

Opposing to previous findings, none of the well-known factors influenced the ability to perform TBF and the performance to adequately answer during this study [9,17-18].

There were some challenges encountered during the course of this study. It was particularly difficult to access to laboratories due to complex paperwork and the laboratory staffs were little motivated to participate in the survey. Also, routine performance of the TBF may have been positively influenced by the presence of investigators in the laboratories; indeed, laboratory staff improved their attention when being assessed on the spot. In all cases, investigators had also checked availability of the written malaria microscopy SOP and the laboratories target were not notified before the assessment day. Finally, the two slides collected for cross-checking did not comply with the WHO recommendations requiring a minimum of 10 slides of which 5 were low parasite density and 5 did not contain parasites [4]. Indeed, in the majority of participating laboratories’ slides were recycled and a TBF preservation system was not implemented at the time of the study.

Satisfactorily, the main strength of this study is real-time assessment of routine TBF, which completes studies recently carried out in the DRC [7,9]. Moreover, data reported from this study will enable health authorities, particularly in the NMCP, to improve the selection of health facilities to be supervised for inclusion in future training sessions. Also, the topics of discussion during training sessions would be adapted to the shortcomings reported in this study [17].

## Conclusions

We successfully established during the course of this study that among clinical laboratories in Kinshasa, the best scores obtained to perform TBF contrasted with poor performance in cross-checking slides. This finding shows that it is imperative to strengthen and standardise periodic formative supervision and cross-checking of slides. This will improve the ability of laboratory staff to perform, read and analyse TBF in order to meet the requirements for accurate results.
